# *In vitro*, *in vivo*, and cellular mechanisms of *Astragalus onobrychis L.* extract against protoscoleces and hydatid cysts of *Echinococcus granulosus*

**DOI:** 10.3389/fphar.2025.1531114

**Published:** 2025-03-24

**Authors:** Javad Ghasemian Yadegari, Amal Khudair Khalaf, Aram Oladi, Ali Shahbazi, Hossein Mahmoudvand

**Affiliations:** ^1^ Department of Pharmacognosy, Lorestan University of Medical Sciences, Khorramabad, Iran; ^2^ Department of Microbiology, College of Medicine, University of Thi-Qar, Thi-Qar, Iraq; ^3^ Deputy of Food and Drug, Lorestan University of Medical Sciences, Khorramabad, Iran; ^4^ Student Research Committee, Lorestan University of Medical Sciences, Khorramabad, Iran; ^5^ Razi Herbal Medicines Research Center, Lorestan University of Medical Sciences, Khorramabad, Iran

**Keywords:** herbal medicines, scolicidal, apoptosis, *Echinococcus granulosus*, hydatidosis

## Abstract

**Introduction:**

In this study, we evaluated the *in vitro, ex vivo*, and *in vivo* effects of the chloroform extract of *Astragalus onobrychis* L. (Fabaceae family) (AOCE) on apoptosis induction and DNA damage in protoscoleces and hydatid cysts of *Echinococcus granulosus*.

**Methods:**

The protoscolicidal properties of AOCE were examined through both *in vitro* and *ex vivo* studies on hydatid cyst protoscoleces, utilizing the eosin exclusion assay. Additionally, we evaluated the effects of AOCE on apoptosis induction and DNA damage in the protoscoleces using a colorimetric protease assay and real-time PCR analysis, respectively. The *in vivo* efficacy was determined by measuring the quantity, dimensions, and mass of hydatid cysts in infected murine subjects.

**Results:**

The findings indicated that AOCE, particularly at a concentration of 45.0 mg/mL, effectively eliminated protoscoleces of hydatid cysts within a 30-min exposure period. Additionally, AOCE demonstrated prolonged anti-parasitic effects in *ex vivo* conditions, in contrast to the immediate lethal effects observed *in vitro* (*p* < *0.001*). AOCE significantly (*p* < *0.01*) induced caspase-3 activation in protoscoleces obtained from hydatid cysts relative to the control normal saline group. Furthermore, the results from Real-time PCR analysis indicated a significant (*p* < *0.001*) upregulation in the expression levels of the *EgATM* and *EgP53* genes following treatment with AOCE. By *in vivo*, we found that treatment with AOCE mainly at 200 mg/kg significantly (*p* < *0.001*) reduced the number, size, and weight of hydatid cyst relative to the control group treated with normal saline group. Biochemical analysis also demonstrated that administration of AOCE to infected mice, led to a marked improvement and a reduction in serum levels of liver function factors.

**Conclusion:**

The results indicated that AOCE exhibits considerable *in vitro* and *ex vivo* scolicidal properties against hydatid cyst protoscoleces. Furthermore, the results highlighted AOCE’s capacity to eradicate protoscoleces through the induction of apoptosis and the infliction of DNA damage. Additionally, AOCE demonstrated significant therapeutic efficacy in managing hydatid cysts in murine models. However, further studies are required to clarify the specific mechanisms underlying its action and to assess its efficacy in clinical trials, which may facilitate the application of AOCE in the context of hydatid cyst surgical procedures.

## 1 Introduction

*Echinococcus granulosus*, a zoonotic cestode, is the causative agent of cystic echinococcosis (CE), commonly known to as hydatid disease (Agudelo Higuita et al., 2016). The prevalence of human CE infections is significant on a global scale, with the majority of cases reported in low- and middle-income countries (Agudelo Higuita et al., 2016). The life cycle of *E. granulosus* involves canid species as definitive hosts, while herbivorous or omnivorous species serve as intermediate hosts. Human infection typically occurs through the accidental ingestion of eggs present in soil, water, or vegetables that have been contaminated with feces from infected canines (Rinaldi et al., 2014). In humans, hydatid cysts are predominantly located in the liver and lungs; however, they may also be found in the abdominal cavity and the nervous system, leading to a diverse array of clinical manifestations (Rinaldi et al., 2014).

Currently, there are several primary treatment modalities for CE, including surgical resection, PAIR (puncture, aspiration, injection, and re-aspiration), and chemotherapy (Velasco-Tirado et al., 2018). Surgical intervention, frequently accompanied by adjuvant chemotherapy, is the predominant treatment approach utilized globally; however, its applicability is limited across different stages of cyst development (Dziri et al., 2009). A notable contributor to recurrence is the rupture of cysts, which results in the release of protoscoleces-rich fluid during surgical procedures (Dziri et al., 2009). To improve the safety of cyst surgery, the application of suitable scolicidal agents is critical. A variety of materials and techniques have been investigated in this regard, many of which are linked to adverse effects. For example, the injection of hypertonic saline, formalin, silver nitrate, and cetrimide into cysts has been reported, yet these agents frequently result in complications such as leakage and necrosis of adjacent healthy tissues (Sharafi et al., 2017). Therefore, the identification of an effective pharmacological agent to reduce the risk of recurrence is of paramount importance.

Medicinal plants and their derivatives are acknowledged as important sources of a wide variety of beneficial therapeutic compounds (Ali et al., 2020). Recent investigations have assessed the antiparasitic properties of certain medicinal plants and their derivatives, including *Pistacia* spp., *Allium* spp, *Curcuma* spp., and *Mentha* spp., through both *in vitro* and *in vivo* studies aimed at *E. granulosus* protoscoleces and hydatid cysts (Ali et al., 2020; Kohansal et al., 2017). Nonetheless, the results of these studies are limited by several factors, including a lack of understanding of the fundamental mechanisms of action and concerns regarding potential toxicity.

The genus *Astragalus* (Fabaceae family) comprises approximately 3,000 species of predominantly perennial herbs, with over 250 recognized taxonomic classifications worldwide (Li et al., 2014). This genus is widely distributed across temperate regions, with around 800 species identified in the pastures and mountainous areas of Iran. Many species within the *Astragalus* genus have a rich history of utilization in traditional medicine for the treatment of various health conditions, such as diabetes, nephritis, gastric ulcers, hypertension, and chronic bronchitis (Shahrajabian et al., 2019). Additionally, a variety of pharmacological properties associated with this genus have been documented, including antioxidant activity, enhancement of the immune system, antihypertensive effects, as well as antimicrobial and anti-inflammatory properties (Shahrajabian et al., 2019; Heinrich et al., 2022). In light of the previously discussed information and the recognized antimicrobial properties of the *Astragalus* genus, along with the scarcity of research concerning *Astragalus onobrychis L.*, the current research seeks to examine the *in vitro, in vivo*, and cellular mechanisms of *A. onobrychis* chloroform extract (AOCE) against protoscoleces and hydatid cysts of *E. granulosus.*

## 2 Materials and methods

### 2.1 Ethical considerations

This project was approved by the ethics committee of Lorestan University of Medical Sciences and with ethics code IR.LUMS.REC.1402.217.

### 2.2 Plant materials

**FIGURE 1:**
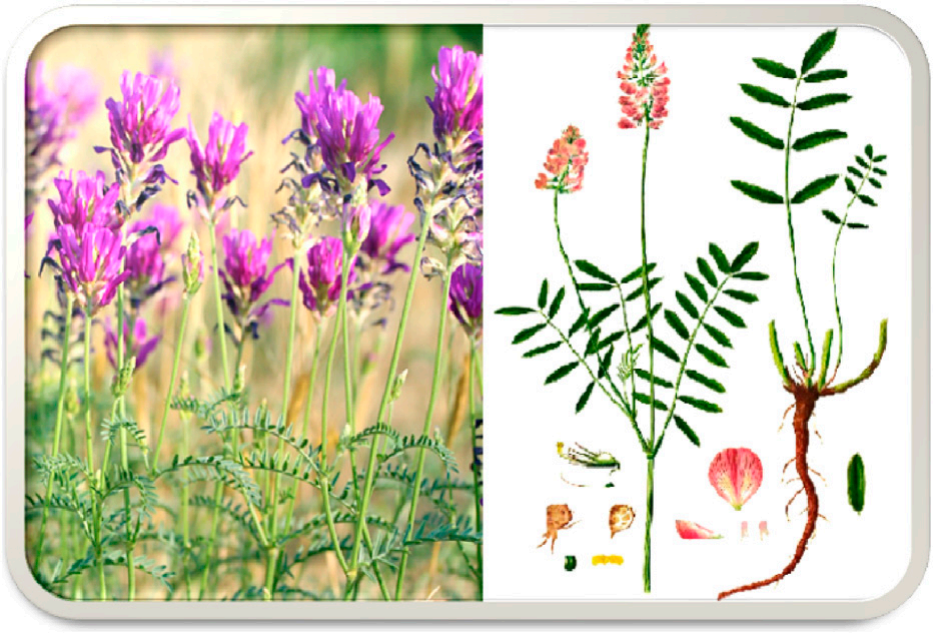
Various parts of *Astragalus onobrychis* belonging to the family Fabaceae.

The root portions of *A. onobrychis* were collected from the rural regions of the Arasbaran area in East Azerbaijan, Iran in May 2022 (Figure 1). Following the identification of the plant specimens by a qualified botanist, a voucher specimen was archived in the herbarium of the School of Pharmacy at Lorestan University of Medical Sciences in Khorramabad, Iran, under the reference number LUMS-26354.

### 2.3 Preparation of the extract

The roots of the plant, which were air-dried and weighed 200 g, were ground into a fine powder. Subsequently, a defatting procedure was carried out using n-hexane, after which extraction was performed through the maceration method utilizing 70% methanol. The resultant methanol extract was concentrated using a rotary evaporator at a temperature of 50°C under vacuum conditions. The concentrated extract was then decanted with chloroform in a decantation funnel and stored at −20°C for subsequent analysis (Mahmoudvand et al., 2016a; Ezatpour et al., 2016).

### 2.4 Phytochemical analysis

The initial phytochemical analysis of the AOCE was conducted to verify the presence of tannins, saponins, alkaloids, flavonoids, and glycosides, in accordance with findings from prior studies (Ezatpour et al., 2016).

#### 2.4.1 Total phenolic

The total phenolic content was determined using the Folin-Ciocalteau colorimetric method, with gallic acid employed as the standard reference (Singleton et al., 1999). In this methodology, 3 mL of Folin-Ciocalteau reagent was mixed with 0.3 mL of the extract and 3 mL of sodium carbonate solution. After a 30-min incubation period in the dark, the absorbance of the resulting mixture was measured at a wavelength of 760 nm using a spectrophotometer. The total phenolic content was reported as milligrams of gallic acid equivalents (GAE) per Gram of dry weight (mg GAE/g DW).

#### 2.4.2 Flavonoid contents

The total flavonoid content was quantified utilizing the aluminum chloride colorimetric method, with quercetin serving as the standard reference (Phuyal et al., 2020). In this procedure, 0.2 mL of the extract or standard solution was combined with 0.2 mL of a 2% aluminum chloride solution and 0.1 mL of a 33% acetic acid solution, followed by thorough mixing. The resulting reaction mixture was then diluted with 90% ethanol to achieve a final volume of 5 mL. The samples were allowed to incubate at room temperature for 30 min. Subsequently, the absorbance was measured at a wavelength of 510 nm using a spectrophotometer, and the findings were expressed as milligrams of quercetin equivalents per Gram of dry weight (mg QE/g DW).

##### 2.4.2.1 Isolation major flavonoids

A 2 g sample was subjected to vacuum liquid chromatography (VLC) utilizing silica gel, with elution conducted using a gradient of chloroform (CHCl_3_): methanol (MeOH) (95:5) to CHCl_3_:MeOH (5:95) in 200 mL increments, followed by an additional 300 mL of MeOH. This procedure yielded a total of 18 fractions, and the entire process was repeated three times to ensure adequate quantities of each fraction. Fraction 5, which measured 178 mg and was eluted with CHCl_3_: MeOH (75:25), produced a mixture of two compounds. These compounds were subsequently purified using Sephadex LH 20 and eluted with MeOH, resulting in the isolation of pure compounds 1 (9 mg) and 2 (24 mg). Fraction 7, weighing 480 mg, underwent partitioning on a Sep-Pak (RP-18, 10 g, Waters, Ireland) with a stepwise gradient of MeOH:H_2_O (ranging from 2:8 to 10:0) in 200 mL increments, yielding five subfractions. Compound 3 was isolated from subfraction two through preparative high-performance liquid chromatography (HPLC), equipped with a diode array detector (DAD) (Shimadzu, LC 20A, Japan), employing acetonitrile (A) and water (B) as eluents. The HPLC was conducted on a C_18_ column (250 × 20 mm, 10 µm) utilizing a gradient elution from 20% to 55% solvent A in solvent B over a 50-min period, with a flow rate of 15 mL/min. Detection was performed at wavelengths of either 260 nm or 330 nm. The peak observed at a retention time of 19.3 min was collected, and the solvent was subsequently removed under vacuum, yielding pure compound 3 (16 mg). Fraction 14, which contained 571 mg, was eluted using a solvent mixture of CHCl_3_:MeOH in a ratio of 30:70 and was identified as a saponin-rich fraction through thin-layer chromatography (TLC) on pre-coated silica gel 60 F254 plates (Merck, Germany), utilizing CHCl_3_:MeOH (13:7) as the eluent. Detection of the compounds was achieved by spraying a 10% ethanolic solution of H_2_SO_4_ on the uncoated areas of the plates (Kavtaradze et al., 2011; Yu et al., 2005; Pu et al., 2015).

### 2.5 Preparation of protoscoleces

Initially, livers harboring cysts are sourced from infected sheep at the Khorramabad slaughterhouse and subsequently transported to the laboratory of the Parasitology Department. Under sterile conditions, the necessary protoscoleces are extracted from the cysts within the liver and placed into sterile tubes. These protoscoleces are then washed with sterile normal saline a minimum of three times (Mahmoudvand et al., 2016b).

### 2.6 Viability assessment

Following the acquisition of protoscoleces, we evaluate their viability percentage through the observation of flame cell motility and staining with 0.1% eosin stain (Sigma-Aldrich, St. Louis, MO, USA). A total of 100 protoscoleces are examined, with those that remain unstained by eosin classified as viable. Only hydatid cysts exhibiting a viability rate exceeding 90% were selected for subsequent testing (Mahmoudvand et al., 2016b).

### 2.7 *In vitro* assessment of the protoscolicidal effect

The *in vitro* protoscolicidal effects of AOCE were estimated at concentrations of 56.25, 112.5, 225, and 450 mg/mL over exposure durations of 5, 10, 20, 30, and 60 min, following established methodologies. Following treatment with the various AOCE concentrations for the specified time intervals, the viability rate of the protoscoleces was assessed using an eosin staining technique. Specifically, 50 μL of 0.1% eosin stain was introduced to the treated protoscoleces, which were then smeared onto a glass slide, covered with a coverslip, and examined under a light microscope. The viability rate was subsequently calculated by counting the number of dead protoscoleces among a total of 300 examined. In this assay, viable protoscoleces appeared colorless and exhibited characteristic muscular movements and flame cell activity, whereas eosin penetrated the dead protoscoleces, rendering them red. Additionally, normal saline served as a negative control, while Ag-nitrate was utilized as a positive control. All experimental procedures are conducted in triplicate using 48-well plates (Mahmoudvand et al., 2019). The 50% inhibitory concentration (IC_50_) is determined through Probit analysis utilizing SPSS software version 25.0.

### 2.8 *Ex vivo* investigation of the protoscolicidal effect

In this phase of the study, the liver of sheep naturally infected with hydatid cysts is employed. Initially, more than 50% of the hydatid fluid is aspirated to isolate protoscoleces, whose viability is subsequently assessed using a 0.1% eosin staining test. For each target concentration of AOCE, three hydatid cysts will be utilized. AOCE will be applied until the entire inner surface of each cyst is thoroughly saturated. Following this, a small volume of cyst fluid will be extracted at predetermined time intervals (7, 10, 12, 15, 20, 25, and 30 min). In the subsequent step, this fluid will be combined with 0.1% eosin. After a period of 10 min, a smear will be prepared from the remaining protoscoleces and placed on a glass slide for examination under a light microscope, with the aim of evaluating the viability of the protoscoleces (Alyousif et al., 2021). The IC_50_ value will be determined using Probit analysis within SPSS software version 25.0.

### 2.9 Assessment of caspase-3-like activity

This investigation employed a colorimetric protease assay, utilizing the Sigma Kit, to assess apoptotic activity through the measurement of caspase-3-like activity in protoscoleces treated with AOCE, following the manufacturer’s protocol. The assay involved monitoring spectrophotometric changes in color, which resulted from the release of a molecule (pNA linked to the substrate) due to the enzymatic activity of caspase-3. After a 48-h exposure of protoscoleces to AOCE, the samples were centrifuged at 600 rpm for 5 min at 4 °C. The sedimented protoscoleces were then lysed and subjected to a second centrifugation at 20,000 rpm for 10 min. In the subsequent procedure, 5 μg of the supernatant was mixed with 85 μL of buffer and 10 μL of the caspase-3 substrate (pNA-DEVD-Ac), and the resulting mixture was incubated for 2 h at 37 °C. The final absorbance of the samples was quantified at 405 nm using an ELISA reader (Cheraghipour et al., 2024).

### 2.10 Effect on the expression of DNA damage related genes

The impact of AOCE on the expression of DNA damage-related genes, specifically ataxia-telangiectasia mutated (*EgATM*) and *EgP53*, in the protoscoleces of *E. granulosus* was assessed using quantitative real-time polymerase chain reaction (qRT-PCR) techniques. Total RNA was extracted in accordance with the manufacturer’s instructions utilizing a commercial kit (Parstous, Iran). The conversion of RNA to complementary DNA (cDNA) was conducted using a kit from Sinaclon Company, adhering to the provided protocols. The primers for *EgATM* (F’: GTT​CCT​ACA​GTC​CAT​CCT​AAT and R’: CTC​CAT​CAA​GCC​AGC​ATT) and *EgP53* (F’: AACCACCGAACTCACAAC and R’: AAC​CGA​CAC​AAC​TCA​TCA​A) were selected based on prior research conducted by Lu et al. (2021). The qRT-PCR protocol initiated with an initial denaturation step at 92°C for 10 min, followed by 40 cycles comprising a denaturation phase at 97°C for 10 s and an annealing/extension phase at 55°C for 40 s. The expression levels of *EgATM* and *EgP53* were quantified using the optical system software (iQTM5 model, Bio-Rad, Hercules, CA) through the application of the 2^−ΔΔCT^ method, with β-actin utilized as the housekeeping gene.

### 2.11 Animals

A total of forty-eight male NMRI mice, aged between 6–7 weeks and weighing between 20 and 25 g, were procured from the Animal House facility at Lorestan University of Medical Sciences, Khorramabad, Iran. The mice were maintained under controlled environmental conditions, which included a temperature of 24°C ± 1°C, a 12-h light/dark cycle, and humidity levels ranging from 40% to 70%. They were given unrestricted access to both food and water.

### 2.12 Hydatid cyst in mice

The establishment of an animal model for hydatid cysts was achieved through the intraperitoneal administration of 500 µL of a solution containing 2,000 protoscoleces. Following a 3-month post-infection period, the presence of hydatidosis in the subjects was confirmed by anesthetizing one animal from each group and visually inspecting the peritoneal cavity for hydatid cysts, as documented by Raziani et al. (2023).

### 2.13 Study design and treatment

Mice were randomly allocated into six experimental groups, each consisting of eight mice including:

i. Non-infected and non-treated mice
ii. Infected mice orally received normal saline for 28 days
iii. Infected mice orally received AOCE at 50 mg/kg/day for 28 days
iv. Infected mice orally received AOCE at 100 mg/kg/day for 28 days
v. Infected mice orally received AOCE at 200 mg/kg/day for 28 days
vi. Infected mice orally received albendazole (AZ) at 200 mg/kg/day for 28 days

### 2.14 Impact of AOCE on hydatid cysts in murine models

In accordance with the established protocol, the experimental mice were subjected to deep euthanasia *via* intraperitoneal injection of a ketamine (100 mg/kg) + xylazine (10 mg/kg). Following this procedure, a meticulous dissection of the peritoneal cavity was conducted to isolate the hydatid cysts from the surrounding tissues. The extracted cysts were subsequently evaluated for their size, quantity, and weight (Raziani et al., 2023). Furthermore, blood samples were obtained from the cardiac region of the mice and subsequently preserved for biochemical analysis.

### 2.15 Impact on hepatic function factors

To evaluate the toxicity levels, serum samples collected from mice in all experimental groups were subjected to analysis utilizing commercially available kits from ParsAzmon, Iran. This assessment was conducted to quantify the concentrations of alanine aminotransferase (ALT), aspartate aminotransferase (AST), total bilirubin (TB), and direct bilirubin (DB), as detailed by Mahmoudvand et al. (2020).

### 2.16 Data analysis

Following the data collection process, descriptive statistics, including the computation of central tendency and dispersion indices, were employed to characterize the dataset. The analysis of variance (ANOVA), along with Tukey’s and *post hoc* tests, was utilized to examine the data further. All statistical analyses were conducted using SPSS version 26 software, with a two-tailed significance level set at *p < 0.05*.

## 3 Results

### 3.1 Phytochemical analysis, total phenolic and flavonoid contents

The chloroform extract produced a yield of 20.1 g, which equates to a concentration of 10.05% (*w/v*). Phytochemical analysis confirmed the presence of saponins, flavonoids, terpenoids, and polysaccharides within the extract. Furthermore, analytical assessments revealed that the total phenolic content was quantified at 0.92 mg GEA/g DW, while the total flavonoid content was determined to be 1.97 mg QE/g DW.

#### 3.1.1 Isolation major flavonoids

Supplementary File S1 is indicated the 1D and 2D NMR spectra of compounds 1-3 in CD3OD purified from *A. onobrychis* extract. Compound 1 was isolated as a white amorphous powder, with its molecular formula determined to be C_15_H_12_O4 through 13C NMR analysis. Based on this comprehensive data and a comparison with previously reported literature values, the structure of compound 1 was confirmed to be liquiritigenin. Compound 2 was identified as a pale-yellow amorphous powder, with its molecular formula established as C_16_H_12_O_4_ based on 13C NMR analysis. The proposed structure of compound 2 corresponds to formononetin, which aligns with existing literature. Compound 3 was isolated as a yellow powder with the molecular formula C_21_H_20_O12, as determined by 13C NMR spectroscopy. The results showed that assignments of all carbon and proton signals were derived from the 2D NMR spectra, leading to the conclusion that the structure of compound 3 is isoquercitrin (quercetin-3-O-glucoside).

### 3.2 *In vitro* investigation of the protoscolicidal effect

**FIGURE 2:**
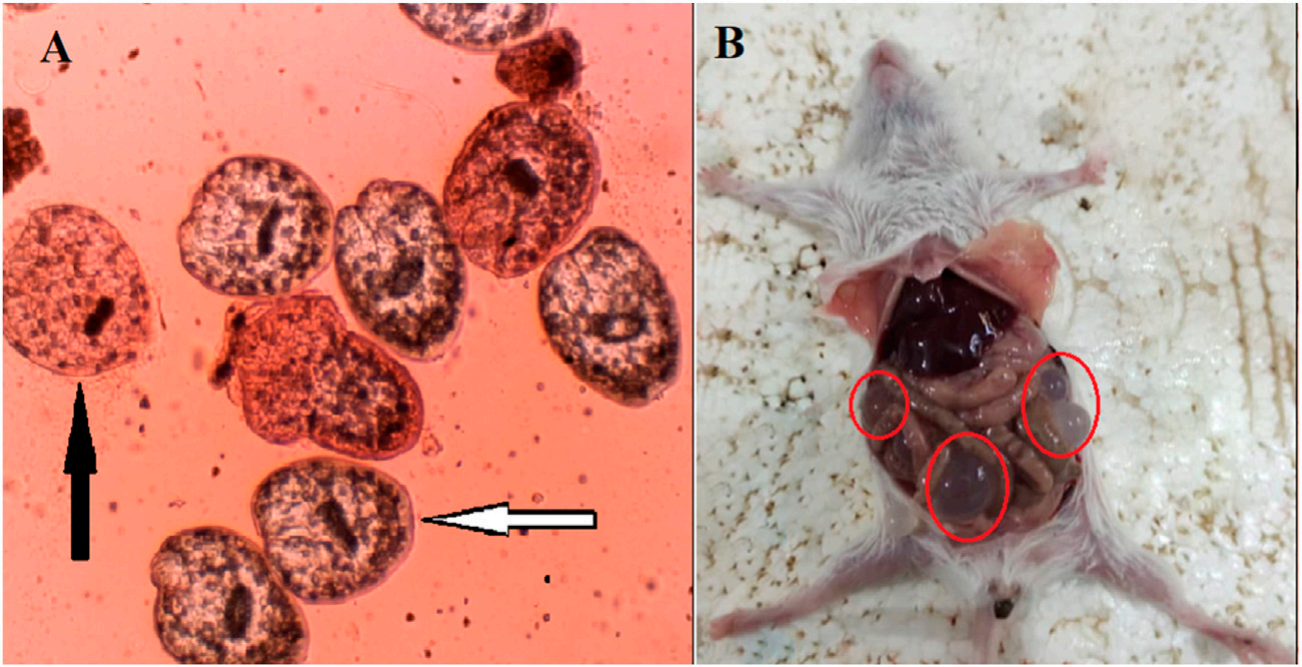
The live (live arrow) and death (black arrow) hydatid cyst protoscoleces **(A)** as well as the hydatid cyst infected mice **(B)** after exposure to *Astragalus onobrychis* chloroform extract by eosin exclusion test.

**FIGURE 3:**
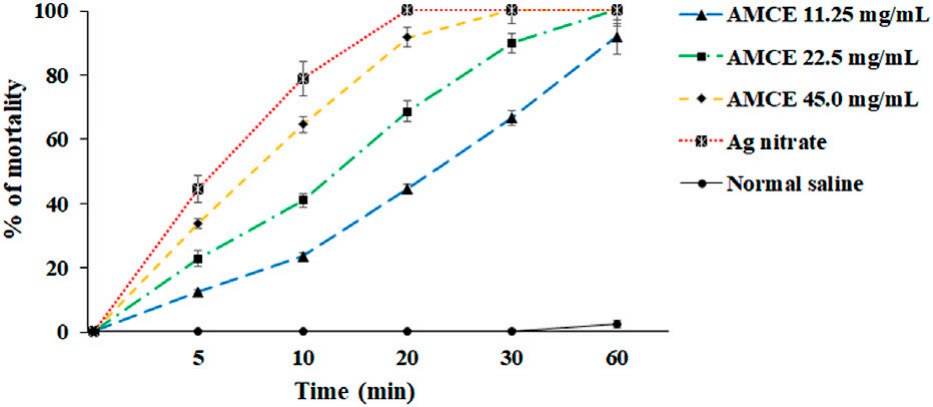
*In vitro* effects of *Astragalus onobrychis* chloroform extract (AOCE) on hydatid cyst protoscoleces after 5, 10, 20, 30, and 60 min exposure time by eosin exclusion assay. (Mean ± SD). n = 3.

The results of the study demonstrate that the AOCE, at a concentration of 45 mg/mL, effectively destroyed protoscoleces of hydatid cysts within a 30-min exposure period (Figure 2A). Conversely, a concentration of 22.5 mg/mL resulted in the complete destruction of protoscoleces after 60 min of exposure. Among the concentrations tested, 11.25 mg/mL exhibited the lowest viability of protoscoleces, leading to an 91.6% reduction after 60 min. Collectively, the findings indicate that AOCE at various concentrations significantly reduced the viability of hydatid cyst protoscoleces (*p < 0.001*) in comparison to the control group (normal saline), as illustrated in Figure 3. The IC_50_ values for AOCE at the time intervals of 5, 10, 20, 30, and 60 min were determined to be > 45.0, 27.71, 12.69, 8.4.8, and 6.14 mg/mL, respectively.

### 3.3 *Ex vivo* investigation of the protoscolicidal effect

**FIGURE 4:**
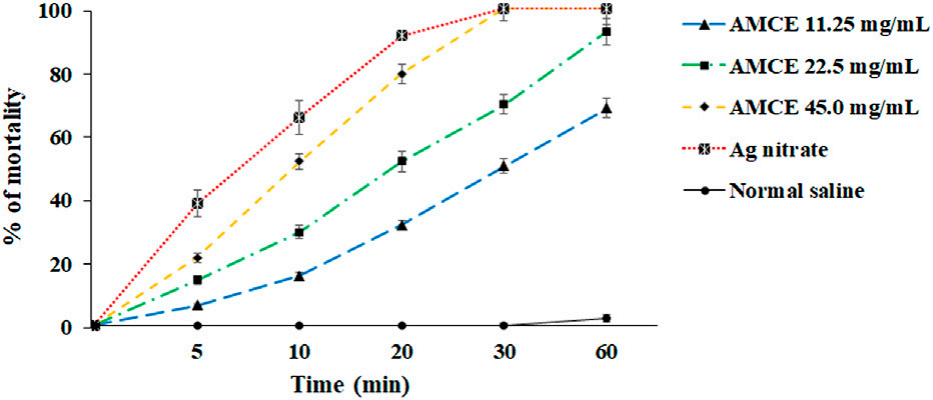
*Ex vivo* effects of various concentrations of *Astragalus onobrychis* chloroform extract (AOCE) on hydatid cyst protoscoleces after 5, 10, 20, 30, and 60 min exposure time by eosin exclusion assay. (Mean ± SD). n = 3.

The results of this investigation indicate that AOCE exhibits extended anti-parasitic effects in an *ex vivo* context, in contrast to its lethal effects observed *in vitro*. Notably, at a concentration of 45.0 mg/mL, AOCE was effective in completely eradicating hydatid cyst protoscoleces following a 30-min exposure. Conversely, a concentration of 22.5 mg/mL resulted in the destruction of 92.7% of protoscoleces after a 60-min exposure. Consistent with the *in vitro* findings, AOCE at 11.25 mg/mL displayed the lowest protoscolicidal activity among the concentrations evaluated, achieving a 68.4% reduction in protoscoleces after 60 min. Additionally, the data revealed that AOCE, at concentrations of 11.25, 22.5, and 45.0 mg/mL, significantly (*p < 0.001*) induced mortality in protoscoleces derived from hydatid cysts in an *ex vivo* setting when compared to the control group (Figure 4). The IC_50_ values for CV at time intervals of 5, 10, 20, 30, and 60 min were determined to be > 45.0, 36.52, 17.86, 11.21, and 8.22 mg/mL, respectively.

### 3.4 Evaluating the caspase-3-like activity

Table 1 presents the impact of different concentrations of AOCE on caspase-3-like activity in protoscoleces derived from hydatid cysts. The results indicate that AOCE induced caspase-3 activation in protoscoleces obtained from hydatid cysts in a dose-dependent manner, particularly at concentrations of 1/2 IC_50_ and IC_50_. This increase in activation of the caspase-3 enzyme is significant relative to the control normal saline group. (*p < 0.001*).

**TABLE 1:** Evaluating the caspase-3-like activity of hydatid cyst protoscoleces after exposure to various concentrations of *Astragalus onobrychis* chloroform extract (AOCE) by the colorimetric protease assay. (Mean ± SD). IC50: The 50% inhibitory concentration. (n = 3).

Drug	% Of caspase-3 activity (Mean ± SD)
AOCE 1/4 IC50	3.9 ± 1.15
AOCE 1/3 IC50	5.6 ± 2.05
AOCE 1/2 IC50	18.6 ± 2.51*
AOCE IC50	26.1 ± 3.15*
Normal saline	2.8 ± 1.66

### 3.5 Effect on the expression of DNA damage related genes

**FIGURE 5:**
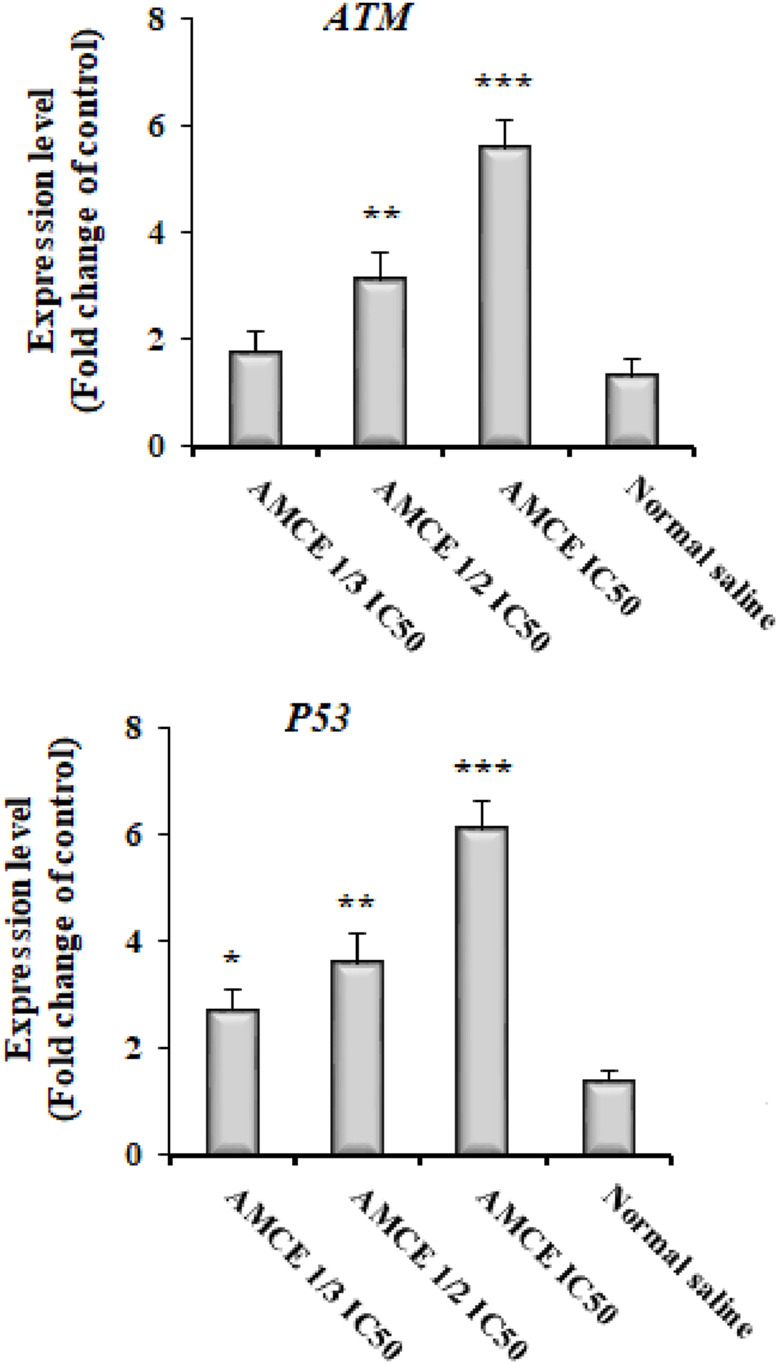
Effect of various concentrations of *Astragalus onobrychis* chloroform extract (AOCE) on the expression of DNA damage related genes of *EgATM* and *EgP53* by Real-time PCR. (Mean ± SD). IC50: The 50% inhibitory concentration. (n = 3).

The results obtained from the Real-time PCR analysis indicated a notable upregulation in the expression levels of the *EgATM* and *EgP53* genes (Figure 5). Specifically, the expression levels of *EgATM* gene increased by 2.71-, 3.59-, and 6.11-fold change following treatment with AOCE at concentrations of 1/3 IC_50_, 1/2 IC_50_, and IC_50_, respectively. Where, the expression levels of *EgP53* gene increased by 1.79-, 3.11-, and 5.59-fold change following treatment with AOCE at concentrations of 1/3 IC_50_, 1/2 IC_50_, and IC_50_, respectively. These variations were found to be statistically significant when compared to the control group (*p < 0.001*).

### 3.6 *In vivo* effects of AOCE on hydatid cyst in mice

*In vivo* investigations into the effects of AOCE on hydatid cysts in murine models revealed that these cysts were predominantly located in the liver and peritoneal cavity (Figure 2B). The negative control group, which was administered normal saline, displayed the highest incidence (11.62 ± 1.18 cysts), largest dimensions (7.12 ± 0.83 mm), and greatest weight of hydatid cysts (279.25 ± 19.18 mg) (Table 2). Conversely, treatment with AOCE dose-dependently reduced the number, size, and weight of hydatid cyst compared with the control normal saline group. The highest efficacy was observed in AOCE at 200 mg/kg which resulted in the maximum reduction (*p < 0.001*) in the number (1.87 ± 0.64 cysts), size (2.25 ± 0.70 mm), and weight (68.63 ± 7.93 mg) of hydatid cysts.

**TABLE 2:** *In vivo* effects of *Astragalus onobrychis* chloroform extract (AOCE) and albendazole (AZ) on the number, size, and weight of hydatid cysts in the infected mice. *p < 0.05; **p < 0.001, ***p < 0.001 compared with control normal saline group. The data are expressed as Mean ± Standard Deviation.

Drug	Number of cysts	Size of cysts (mm)	Weight of cysts (mg)
Normal saline	11.62 ± 1.18	7.12 ± 0.83	279.25 ± 19.18
AOCE 50 mg/kg	7.6 ± 0.74*	5.12 ± 0.64*	180.82 ± 18.82*
AOCE 100 mg/kg	5.37 ± 0.91**	3.0 ± 0.92**	119.66 ± 13.11***
AOCE 200 mg/kg	1.87 ± 0.64***	2.25 ± 0.70***	68.63 ± 7.93***
AZ 200 mg/kg	2.12 ± 0.64***	2.62 ± 0.74***	74.5 ± 4.21***

### 3.7 Effect of AOCE on liver function factors

The results of the biochemical analyses demonstrated a significant elevation in serum levels of ALT, AST, and TB in the infected mice infected with hydatid cyst (*p < 0.001*). In contrast, the administration of AOCE to infected mice, at dosages of 50, 100, and 200 mg/kg, led to a marked improvement and a reduction in serum levels of ALT, AST, and TB (*p < 0.001*) (Table 3).

**TABLE 3:** Effects of *Astragalus onobrychis* chloroform extract (AOCE) and albendazole (AZ) on the liver function factors of alanine aminotransferase (ALT), aspartate aminotransferase (AST), and total bilirubin (TB) in mice with hydatid cyst. *p < 0.05; **p < 0.001, ***p < 0.001 compared with control normal saline group. The data are expressed as Mean ± Standard Deviation.

Drug	ALT (IU/L)	AST (IU/L)	TB (mg/dL)
Normal saline	178.66 ± 14.6	234.33 ± 19.66	12.25 ± 2.18
AOCE 50 mg/kg	137.46 ± 13.24*	189.64 ± 16.65*	7.66 ± 1.15*
AOCE 100 mg/kg	109.56 ± 7.92**	154.62 ± 8.86**	5.36 ± 1.15**
AOCE 200 mg/kg	59.45 ± 5.32***	113.66 ± 7.33***	2.23 ± 0.56***
AZ 200 mg/kg	52.33 ± 6.12***	108.33 ± 6.90***	1.96 ± 0.43***
Healthy mice	45.83 ± 3.35	105.33 ± 7.66	1.46 ± 0.36

## 4 Discussion

Surgery, often supplemented by adjuvant chemotherapy, constitutes the primary therapeutic approach for hydatid disease globally, particularly in the management of hydatid cysts; however, this treatment modality is not universally applicable to all stages of cyst development (Dziri et al., 2009). In the context of hydatid cyst surgery, the utilization of appropriate scolicidal agents is essential to enhance the safety of the surgical procedure. A range of materials and techniques has been explored for this purpose, many of which are associated with adverse effects (Sharafi et al., 2017). For instance, the use of hypertonic saline, formalin, silver nitrate, and cetrimide within cysts has been documented, yet these agents often lead to complications such as leakage and necrosis of surrounding healthy tissues (Sharafi et al., 2017).

The aim of this research was to assess the protoscolicidal effects, induction of apoptosis, and DNA damage induced by AOCE on hydatid cyst protoscoleces under both *in vitro* and *ex vivo* conditions. The findings of the *in vitro* indicated that AOCE, particularly at a concentration of 45 mg/mL, effectively eliminated protoscoleces of hydatid cysts within a 30-min exposure period. Additionally, AOCE demonstrated prolonged anti-parasitic activity in *ex vivo* conditions, resulting in the complete eradication of hydatid cyst protoscoleces, in contrast to the immediate lethal effects observed *in vitro*. By *in vivo*, we found that treatment with AOCE mainly at 200 mg/kg significantly reduced the number, size, and weight of hydatid cyst compared with the control normal saline group in the infected mice.

Numerous studies have investigated the anthelmintic and antiparasitic properties of Fabaceae plants, including *Acacia farnesiana* pods, which have been evaluated for their efficacy against *Haemonchus contortus* in female lambs (Olmedo-Juárez et al., 2020). Additionally, *Mucuna pruriens* has been studied for its effects on *Trypanosoma brucei* (Jimoh et al., 2020), *Inga semialata* has been assessed for its activity against *Plasmodium falciparum* (Lima et al., 2022), and *Millettia richardiana* has been examined for its relation to *Leishmania donovani* (Rajemiarimiraho et al., 2014). Research on the antiparasitic properties of various species within the genus *Astragalus* remains limited. Ghasemian Yadegari et al. demonstrated that the extract of *Astragalus maximus* resulted in a statistically significant reduction in the viability of *Giardia lamblia* cysts. Specifically, at concentrations of 22.5 mg/mL and 45 mg/mL, the extract completely eradicated 100% of *G. lamblia* cysts after 2 and 3 h, respectively (Ghasemian Yadegari et al., 2022). In a study conducted by Yang et al., the extract of *Astragalus membranaceus* exhibited effective *in vitro* anti-*Toxoplasma* activity, as indicated by a decrease in the intracellular replication of *Toxoplasma gondii* tachyzoites following 3, 4, and 5 days of incubation (Yang et al., 2012). Furthermore, Abdel-Tawab et al. (2020) reported that *A. membranaceus*, particularly at a concentration of 50 mg/kg, significantly diminished the viability of *Eimeria papillata* oocysts and reduced the average number of oocysts excreted in the feces of infected animals (Abdel-Tawab et al., 2020). Mahmoudvand et al. also found that the extract of *Astragalus ecbatanus* significantly (*p < 0.001*) decreased the viability of both promastigote and amastigote forms of *Leishmania tropica* in comparison to the negative control (Mahmoudvand et al., 2024). Additionally, Ghasemian Yadegari et al. (2022) reported that the extract of *Astragalus brachycalyx* at concentrations of 225 and 450 mg/mL demonstrated potent protoscolicidal effects on hydatid cyst protoscoleces (Yadegari et al., 2022). Mahmoudvand et al. (2022) reported *A. ecbatanus* chloroform extract, particularly at a concentration of 45 mg/mL, exhibited significant anti-helminthic activity against *E. granulosus* protoscoleces; whereas, increased the caspase-3 enzyme activity and the rate of permeability of plasma membrane (Mahmoudvand et al., 2022). Recently, Taheri et al. (2024) showed chloroform extract of *A. ecbatanus* at 10 and 20 mg/kg for 28 days the recovered the lesions of cutaneous leishmaniasis in the infected mice with *Leishmania major* (Taheri et al., 2024). The observed variations in results may be attributed to several factors, including the specific type of parasite, the particular species of *Astragalus* employed, the nature of the extract utilized, the concentration applied, and the methodologies adopted in the studies.

In the preliminary phytochemical analysis, the compounds saponins, flavonoids, terpenoids, and polysaccharides were detected in AOCE. Based on our comprehensive data and a comparison with previously reported literature values, the presence of liquiritigenin, formononetin, and isoquercitrin was confirmed in AOCE. With respect to the antiparasitic effects of these flavonoids’ compounds, de Silva et al. (2012) showed quercetin, quercitrin and isoquercitrin had promising antileishmanial effects against *Leishmania amazonensis* with the IC50 values of 3.8, 10 and 4.3 μM, respectively (da Silva et al., 2012). Haghighi et al. reported (2023) that formononetin, at concentrations of 300 and 150 μg/mL, was found to completely eliminate hydatid cyst protoscoleces after exposure durations of 30 and 60 min, respectively (Haghigh et al., 2023). In addition, Mahmoudvand et al. (2023) exhibited that formononetin displayed potent antiparasitic effects against *L. tropica* and *T. gondii* with the IC50 values of 14.3 and 9.85 μg/mL, respectively (Mahmoudvand et al., 2023a; Mahmoudvand et al., 2023b). Regarding the influence of flavonoid compounds on microbial pathogens, earlier investigations have shown that these compounds exert antimicrobial effects through various mechanisms, including the disruption of the cytoplasmic membrane, inhibition of nucleic acid synthesis, induction of apoptosis, interference with energy metabolism, alteration of membrane permeability, and reduction of pathogenicity (Górniak et al., 2019; Panche et al., 2016). Additionally, polyphenolic compounds are known to possess antimicrobial properties by hindering virulence factors, disrupting cytoplasmic and cell membranes, and inhibiting DNA synthesis (Miklasińska-Majdanik et al., 2018; Othman et al., 2019). Therefore, it can be inferred that the antigiardial activity observed in the extract of AOCE is likely due to the presence of phenolic and flavonoid compounds within the herb.

Apoptosis is acknowledged for its dual role in the interaction between the host and hydatid cysts, contributing to both survival and suppression mechanisms (Paredes et al., 2007). Key enzymes involved in the mediation of apoptosis, such as caspase-3 and caspase-9, play a crucial role in the progression of this process, particularly in relation to DNA fragmentation and the morphological alterations that define cellular death (Paredes et al., 2007). The current findings demonstrate that AOCE dose-dependently (mainly at concentrations of 1/2 IC_50_, and IC_50_) elicited caspase-3 activation levels in protoscoleces derived from hydatid cysts.

DNA damage is identified by proteins that exhibit both signaling and repair capabilities (Žgur-Bertok, 2013). A crucial element of the DNA damage response is the ataxia-telangiectasia mutated (ATM) protein (Maréchal and Zou, 2013). ATM has the ability to detect DNA damage and can trigger G1/S cell cycle arrest and apoptosis by stabilizing the P53 protein (Maréchal and Zou, 2013). RAD54 is an essential compound of the enzymatic machinery responsible for the repair of DNA double-strand breaks (Mazin et al., 2010). Studies have demonstrated that monoterpene compounds can induce DNA damage in microbial pathogens (Nikolić et al., 2019). The results obtained from the Real-time PCR analysis indicated a notable upregulation in the expression levels of the *EgATM* and *EgP53* genes following treatment with AOCE at concentrations of 1/3 IC_50_, 1/2 IC_50_, and IC_50_. The findings indicate that AOCE has been demonstrated to cause DNA damage in protoscoleces and to activate the ATM-P53-Topo2a signaling pathway.

The evaluation of the toxicological characteristics of novel agents is an essential and standard procedure prior to their market release (Mahmoudvand et al., 2020). Biochemical analysis results demonstrated that administration of AOCE to infected mice, at dosages of 50, 100, and 200 mg/kg, led to a marked improvement and a reduction in serum levels of ALT, AST, and TB. These findings suggest that the administration of AOCE at doses ranging from 50 to 200 mg/kg displayed hepatoprotective effects on liver function in mice with hydatid cyst. Future directions of this research may include an examination of the antiparasitic properties of the active constituents of this plant, specifically liquiritigenin, formononetin, and isoquercitrin, against various developmental stages of the parasite *E. granulosus* (e.g., protoscoleces and hydatid cysts), along with an exploration of their potential mechanisms of action.

## 5 Conclusion

The results indicated that AOCE exhibits considerable *in vitro* and *ex vivo* scolicidal properties against hydatid cyst protoscoleces. Furthermore, the results highlighted AOCE’s capacity to eradicate protoscoleces through the induction of apoptosis and the infliction of DNA damage. Additionally, AOCE demonstrated significant therapeutic efficacy in managing hydatid cysts in murine models with potent hepatoprotective effects. However, further studies are required to clarify the specific mechanisms underlying its action and to assess its efficacy in clinical trials, which may facilitate the application of AOCE in the context of hydatid cyst surgical procedures.

## Data Availability

The original contributions presented in the study are included in the article/Supplementary Material, further inquiries can be directed to the corresponding author.

## References

[B1] Abdel-Tawab H. Abdel-Baki A. S. El-Mallah A. M. Al-Quraishy S. Abdel-Haleem H. M. (2020). *In vivo* and *in vitro* anticoccidial efficacy of Astragalus membranaceus against Eimeria papillata infection. J. King Saud University-Science 32 (3), 2269–2275. 10.1016/j.jksus.2020.03.016

[B2] Agudelo Higuita N. I. Brunetti E. McCloskey C. (2016). Cystic echinococcosis. J. Clin. Microbiol. 54 (3), 518–523. 10.1128/JCM.02420-15 26677245 PMC4767951

[B3] Ali R. Khan S. Khan M. Adnan M. Ali I. Khan T. A. (2020). A systematic review of medicinal plants used against Echinococcus granulosus. Plos one 15 (10), e0240456. 10.1371/journal.pone.0240456 33048959 PMC7553295

[B4] Alyousif M. S. Al-Abodi H. R. Almohammed H. Alanazi A. D. Mahmoudvand H. Shalamzari M. H. (2021). Chemical composition, apoptotic activity, and antiparasitic effects of Ferula macrecolea essential oil against Echinococcus granulosus protoscoleces. Molecules 26 (4), 888. 10.3390/molecules26040888 33567639 PMC7914769

[B8] Cheraghipour K. Shakib P. Khalaf A. K. Mehrnia M. Moridnia A. Amiri S. (2024). Unveiling therapeutic effects of *Thymbra spicata* L. on Cystic Echinococcosis. Jundishapur. J. Nat. Pharm. Prod. 19 (2), e146063. 10.5812/jjnpp-146063

[B5] da Silva E. R. do Carmo Maquiaveli C. Magalhães P. P. (2012). The leishmanicidal flavonols quercetin and quercitrin target Leishmania (Leishmania) amazonensis arginase. Exp. Parasitol. 130 (3), 183–188. 10.1016/j.exppara.2012.01.015 22327179

[B6] Dziri C. Haouet K. Fingerhut A. Zaouche A. (2009). Management of cystic echinococcosis complications and dissemination: where is the evidence? World J. Surg. 33, 1266–1273. 10.1007/s00268-009-9982-9 19350321

[B7] Ezatpour B. Azami M. Motamedi M. Rashidipour M. Mahmoudvand H. Alirezaei M. (2016). Chemical composition, *in vitro* antibacterial and cytotoxicity effect of nectaroscordum tripedale extract. J. Herb. Med. J. 1, 29–36.

[B9] Ghasemian Yadegari J. Khudair Khalaf A. Darabi R. (2022). Antiparasitic effects and cellular mechanism of Astragalus maximus chloroform extract against clinical isolates of giardia lamblia. Res. J. Pharmacogn 9, 5–13. 10.22127/rjp.2022.330464.1849

[B10] Górniak I. Bartoszewski R. Króliczewski J. (2019). Comprehensive review of antimicrobial activities of plant flavonoids. Phytochem. Rev. 18 (1), 241–272. 10.1007/s11101-018-9591-z

[B11] Haghigh D. Mahmoudvand H. Khudair Khalaf A. Adineh A. Maleki A. M. Ghasemian Yadegari J. (2023). Antiparasitic effects and cellular mechanisms of formononetin (a natural isoflavone) against hydatid cyst protoscoleces. Jundishapur J. Nat. Pharm. Prod. 18. 10.5812/jjnpp-129302

[B12] Heinrich M. Jalil B. Abdel-Tawab M. Echeverria J. Kulić Ž. McGaw L. J. (2022). Best practice in the chemical characterisation of extracts used in pharmacological and toxicological research—the ConPhyMP—guidelines. Front. Pharmacol. 13, 953205. 10.3389/fphar.2022.953205 36176427 PMC9514875

[B13] Jimoh M. A. Idris O. A. Jimoh M. O. (2020). Cytotoxicity, phytochemical, antiparasitic screening, and antioxidant activities of Mucuna pruriens (Fabaceae). Plants 9 (9), 1249. 10.3390/plants9091249 32971828 PMC7569803

[B14] Kavtaradze N. S. Alaniya M. Mshvildadze V. Skhirtladze A. Lavoie S. Pichette A. (2011). Flavonoids from Astragalus microcephalus. Chem. Nat. Compd. 46 (6), 971–973. 10.1007/s10600-011-9800-0

[B15] Kohansal M. H. Nourian A. Rahimi M. T. Daryani A. Spotin A. Ahmadpour E. (2017). Natural products applied against hydatid cyst protoscolices: a review of past to present. Acta trop. 176, 385–394. 10.1016/j.actatropica.2017.09.013 28935552

[B16] Li X. Qu L. Dong Y. Han L. Liu E. Fang S. (2014). A review of recent research progress on the Astragalus genus. Molecules 19 (11), 18850–18880. 10.3390/molecules191118850 25407722 PMC6270929

[B17] Lima N. M. de Marqui S. R. Andrade T. D. Silva D. H. (2022). Phytochemical, metabolic profiling and antiparasitic potential from Inga semialata leaves (Fabaceae). Nat. Prod. Res. 36 (7), 1898–1903. 10.1080/14786419.2020.1817918 32901524

[B18] Lu S. Wen L. Gong Y. Tian C. Gao H. Chen B. (2021). *In vitro* effects of harmine against Echinococcus granulosus protoscoleces by stimulating DNA damage. Exp. Parasitol. 226, 108121. 10.1016/j.exppara.2021.108121 34097889

[B19] Mahmoudvand H. Al-Abodi H. R. Zolfagharkhani P. Ghasemian Yadegari J. (2022). Anti-helminthic effects and cellular mechanisms of Astragalus ecbatanus extract against Echinococcus granulosus protoscoleces. J. Parasit. Dis. 46 (4), 1047–1054. 10.1007/s12639-022-01517-y 36457771 PMC9606165

[B20] Mahmoudvand H. Khalaf A. K. Rajabi P. Z. Karbasian N. Ghasemian Yadegari J. (2023a). Leishmanicidal and immunomodulatory activities of the formononetin (a natural isoflavone) against Leishmania tropica. BMC Res. Notes 16 (1), 120. 10.1186/s13104-023-06403-1 37365655 PMC10294385

[B21] Mahmoudvand H. Kheirandish F. Ghasemi Kia M. Tavakoli Kareshk A. Yarahmadi M. (2016a). Chemical composition, protoscolicidal effects and acute toxicity of Pistacia atlantica Desf. fruit extract. Nat. Prod. Res. 30 (10), 1208–1211. 10.1080/14786419.2015.1046868 26252652

[B22] Mahmoudvand H. Khudair Khalaf A. Karbasian N. Masoori L. Zareh Rajabi P. Saki M. (2023b). Promising effects of formononetin, a natural isoflavone derived from herbs, against Toxoplasma gondii. J. Herbmed Pharmacol. 12, 362–366. 10.34172/jhp.2023.39

[B23] Mahmoudvand H. Khudair Khalaf A. Masoori L. Ghasemian Yadegari J. (2024). Antileishmanial, immune modulation and apoptosis induction by Astragalus ecbatanus extract against Leishmania tropica. Jundishapur J. Nat. Pharm. Prod. 19 (3), e146164. 10.5812/jjnpp-146164

[B24] Mahmoudvand H. Pakravanan M. Aflatoonian M. R. Khalaf A. K. Niazi M. Mirbadie S. R. (2019). Efficacy and safety of Curcuma longa essential oil to inactivate hydatid cyst protoscoleces. BMC complementary Altern. Med. 19, 187–7. 10.1186/s12906-019-2527-3 PMC6660933 31349828

[B25] Mahmoudvand H. Pakravanan M. Kheirandish F. Jahanbakhsh S. Sepahvand M. Niazi M. (2020). Efficacy and safety Curcuma zadoaria L. To inactivate the hydatid cyst protoscoleces. Curr. Clin. Pharmacol. 15 (1), 64–71. 10.2174/1574884714666190918155147 31533603 PMC7366002

[B26] Mahmoudvand H. Tavakoli Oliaei R. Mirbadie S. R. Kheirandish F. Tavakoli Kareshk A. Ezatpour B. (2016b). Efficacy and safety of Bunium persicum (Boiss) to inactivate protoscoleces during hydatid cyst operations. Surg. Infect. 17 (6), 713–719. 10.1089/sur.2016.010 27501060

[B27] Maréchal A. Zou L. (2013). DNA damage sensing by the ATM and ATR kinases. Cold Spring Harb. Perspect. Biol. 5 (9), a012716. 10.1101/cshperspect.a012716 24003211 PMC3753707

[B28] Mazin A. V. Mazina O. M. Bugreev D. V. Rossi M. J. (2010). Rad54, the motor of homologous recombination. DNA repair 9 (3), 286–302. 10.1016/j.dnarep.2009.12.006 20089461 PMC2827677

[B29] Miklasińska-Majdanik M. Kępa M. Wojtyczka R. D. Idzik D. Wąsik T. J. (2018). Phenolic compounds diminish antibiotic resistance of *Staphylococcus aureus* clinical strains. Int. J. Environ. Res. Public Health 15 (10), 2321. 10.3390/ijerph15102321 30360435 PMC6211117

[B30] Nikolić B. Mitić-Ćulafić D. Vuković-Gačić B. Knežević-Vukčević J. (2019). Plant mono-terpenes camphor, eucalyptol, thujone, and DNA repair. Handb. Nutr. Diet, Epigenetics 1007, 978–983.

[B31] Olmedo-Juárez A. Zarza-Albarran M. A. Rojo-Rubio R. Zamilpa A. González-Cortazar M. Mondragón-Ancelmo J. (2020). Acacia farnesiana pods (plant: Fabaceae) possesses anti-parasitic compounds against *Haemonchus contortus* in female lambs. Exp. Parasitol. 218, 107980. 10.1016/j.exppara.2020.107980 32877640

[B32] Othman L. Sleiman A. Abdel-Massih R. M. (2019). Antimicrobial activity of polyphenols and alkaloids in middle eastern plants. Front. Microbiol. 10, 911. 10.3389/fmicb.2019.00911 31156565 PMC6529554

[B33] Panche A. N. Diwan A. D. Chandra S. R. (2016). Flavonoids: an overview. J. Nutr. Sci. 5, e47. 10.1017/jns.2016.41 28620474 PMC5465813

[B34] Paredes R. Jimenez V. Cabrera G. Iragüen D. Galanti N. (2007). Apoptosis as a possible mechanism of infertility in Echinococcus granulosus hydatid cysts. J. Cell. Biochem. 100 (5), 1200–1209. 10.1002/jcb.21108 17031852

[B35] Phuyal N. Jha P. K. Raturi P. P. Rajbhandary S. (2020). Total phenolic, flavonoid contents, and antioxidant activities of fruit, seed, and bark extracts of Zanthoxylum armatum DC. Sci. World J. 2020, 8780704. 10.1155/2020/8780704 PMC7102453 32256249

[B36] Pu W. Wang D. Zhou D. (2015). Structural characterization and evaluation of the antioxidant activity of phenolic compounds from Astragalus taipaishanensis and their structure-activity relationship. Sci. Rep. 5, 13914. 10.1038/srep13914 26350974 PMC4563559

[B37] Rajemiarimiraho M. Banzouzi J. T. Nicolau-Travers M. L. Ramos S. Cheikh-Ali Z. Bories C. (2014). Antiprotozoal activities of Millettia richardiana (Fabaceae) from Madagascar. Molecules 19 (4), 4200–4211. 10.3390/molecules19044200 24705564 PMC6271796

[B38] Raziani Y. Cheraghipour K. Shakibaie M. Yadegari J. G. Mahmoudvand H. (2023). High potency of magnetic iron oxide nanoparticles covered by piroctone olamine against cystic echinococcosis. Biomed. & Pharmacother. 161, 114536. 10.1016/j.biopha.2023.114536 36940617

[B39] Rinaldi F. Brunetti E. Neumayr A. Maestri M. Goblirsch S. Tamarozzi F. (2014). Cystic echinococcosis of the liver: a primer for hepatologists. World J. hepatology 6 (5), 293–305. 10.4254/wjh.v6.i5.293 PMC4033287 24868323

[B40] Shahrajabian M. H. Sun W. Cheng Q. (2019). A review of Astragalus species as foodstuffs, dietary supplements, a traditional Chinese medicine and a part of modern pharmaceutical science. Appl. Ecol. Environ. Res. 17 (6), 13371–13382. 10.15666/aeer/1706_1337113382

[B41] Sharafi S. M. Sefiddashti R. R. Sanei B. Yousefi M. Darani H. Y. (2017). Scolicidal agents for protoscolices of Echinococcus granulosus hydatid cyst: review of literature. J. Res. Med. Sci. 22 (1), 92. 10.4103/jrms.JRMS_1030_16 28900448 PMC5583616

[B42] Singleton V. L. Orthofer R. LamuelaRaventós R. M. (1999). Analysis of total phenols and other oxidation substrates and antioxidants by means of Folin-Ciocalteu reagent. Methods Enzymol. 299, 152–178.

[B43] Taheri S. N. Mahmoudvand H. Yadegari J. G. Pourhossein S. Masoori L. (2024). *In vitro* and *in vivo* effects of Astragalus ecbatanus extract against cutaneous leishmaniasis. Archives Razi Inst. 79 (5). 10.32592/ARI.2024.79.5.929 PMC12018737 40292066

[B44] Velasco-Tirado V. Alonso-Sardón M. Lopez-Bernus A. Romero-Alegría Á. Burguillo F. J. Muro A. (2018). Medical treatment of cystic echinococcosis: systematic review and meta-analysis. BMC Infect. Dis. 18, 306–309. 10.1186/s12879-018-3201-y 29976137 PMC6034244

[B45] Yadegari J. G. Khalaf A. K. Saadatmand M. Mahmoudvand H. (2022). Antiparasitic activity of *Astragalus brachycalyx* subsp. brachycalyx extract against hydatid cyst protoscoleces and its effect on induction of apoptosis: an *in vitro* and *ex vivo* study. J. Herbmed Pharmacol. 11 (3), 428–434. 10.34172/jhp.2022.49

[B46] Yang X. Huang B. Chen J. Huang S. Zheng H. Lun Z. R. (2012). *In vitro* effects of aqueous extracts of Astragalus membranaceus and Scutellaria baicalensis GEORGI on Toxoplasma gondii. Parasitol. Res. 110 (6), 2221–2227. 10.1007/s00436-011-2752-2 22179265

[B47] Yu D. Bao Y. Wei C. An L. (2005). Studies of chemical constituents and their antioxidant activities from Astragalus mongholicus Bunge. Biomed. Environ. Sci. 18 (5), 297–301. 16370311

[B48] Žgur-Bertok D. (2013). DNA damage repair and bacterial pathogens. PLoS Pathog. 9 (11), e1003711. 10.1371/journal.ppat.1003711 24244154 PMC3820712

