# Supplementary material for: In vitro, in vivo, and cellular mechanisms of Astragalus onobrychis L. extract against protoscoleces and hydatid cysts of Echinococcus granulosus
Source: Front Pharmacol. 2025 Mar 24;16:1531114. doi: 10.3389/fphar.2025.1531114 (PMC11973518; doi:10.3389/fphar.2025.1531114)
Supplement: Supplementary file 1 [file Supplementaryfile1.docx]

**Supplementary file 1A**. One-dimensional (1D) and two-dimensional (2D) nuclear magnetic resonance (NMR) spectra of compounds 1-3, obtained in CD3OD and purified from *Astragalus onobrychis L.*; chemical shifts (δ) are reported in parts per million (ppm) and coupling constants (J) in hertz (Hz).

| **Location** | **Compound 1** | | **Compound 2** | | **Compound 3** | |
| --- | --- | --- | --- | --- | --- | --- |
|  | **δH** | **δC** | **δH** | **δC** | **δH** | **δC** |
| 2 | 5.40 *dd* (J=2.8, 13.2) | 79.65 | 8.19s | 153.44 | - | 157.10 |
| 2a  3b | 2.71 *dd* (J=2.8, 17.2  3.08 dd (J=13.2,17.2) | 43.55 | - | - | - | 134.2 |
| 5 | 7.75 *d* (J=8.8) | 128.48 | 8.09 *d* (J=8.8) | 124.30 | - | 161.63 |
| 6 | 6.52 *dd* (J=1.2, 8.8) | 110.40 | 6.97 *dd* (J=2.4, 8.8) | 115.14 | 6.22 *d* (J=2.4) | 98.62 |
| 7 | - | 165.48 | - | 163.43 | - | 165 |
| 8 | 6.37 *d* (J=1.2) | 102.44 | 6.88 *d* (J=2.4) | 101.86 | 6.39 *d* (J=2.4) | 93.4 |
| 9 | - | 164.19 | - | 158.44 | 157.58 |  |
| 10 | - | 113.55 | - | 116.76 | 104.19 |  |
| 1’ | - | 129.95 | - | 124 | - | 121.66 |
| 2’ | 7.35 *d* (J=8.4) | 127.63 | 7.49 *d* (J=8.6) | 129.99 | 7.74 *d* (J=2.4) | 116.13 |
| 3’ | 6.84 *d* (J=8.4) | 114.93 | 7.02 *d* (J=8.6) | 113.44 | - | 144.53 |
| 4' | - | 157.57 | - | 159.74 | - | 148.48 |
| 4΄ OCH3 | - | - | 3.85 | 54.3 | - | - |
| 5' | 6.84 *d* (J=8.4) | 114.93 | 7.02 *d* (J=8.6) | 113.44 | 6.89 *d* (J=4.8) | 114.61 |
| 6’ | 7.35 *d* (J=8.4) | 127.63 | 7.49 *d* (J=8.6) | 129.99 | 7.62 *dd* (J=2.4,4.8) | 121.80 |
| -  C=O | - | 192.19 | - | 176.68 | - | 176.08 |
| 3 o β  glucopyranosyl |  |  |  |  |  |  |
| 1 |  |  |  |  | 5.27 *d* (J=7.6) | 102.90 |
| 2 |  |  |  |  | 3.5 | 74.31 |
| 3 |  |  |  |  | 3.45 | 76.70 |
| 4 |  |  |  |  | 3.35 t | 69.80 |
| 5 |  |  |  |  | 3.25 m | 76.99 |
|  |  |  |  |  | 3.59 *dd*, 3.74 *dd* | 61.12 |


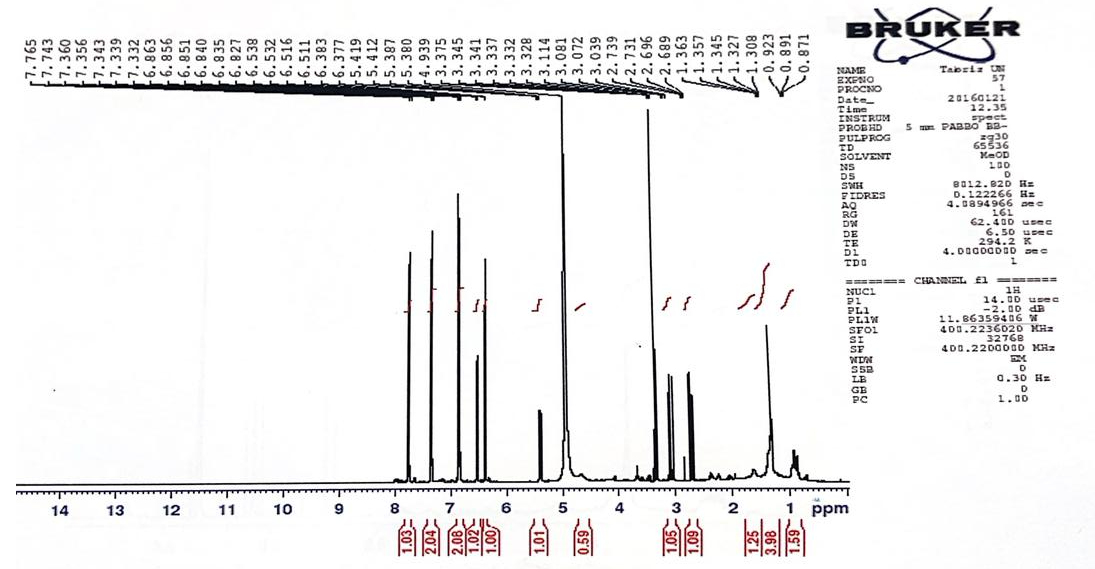


**Supplementary file 1B**. Nuclear magnetic resonance (NMR) spectra of liquiritigenin purified from *Astragalus onobrychis* L.


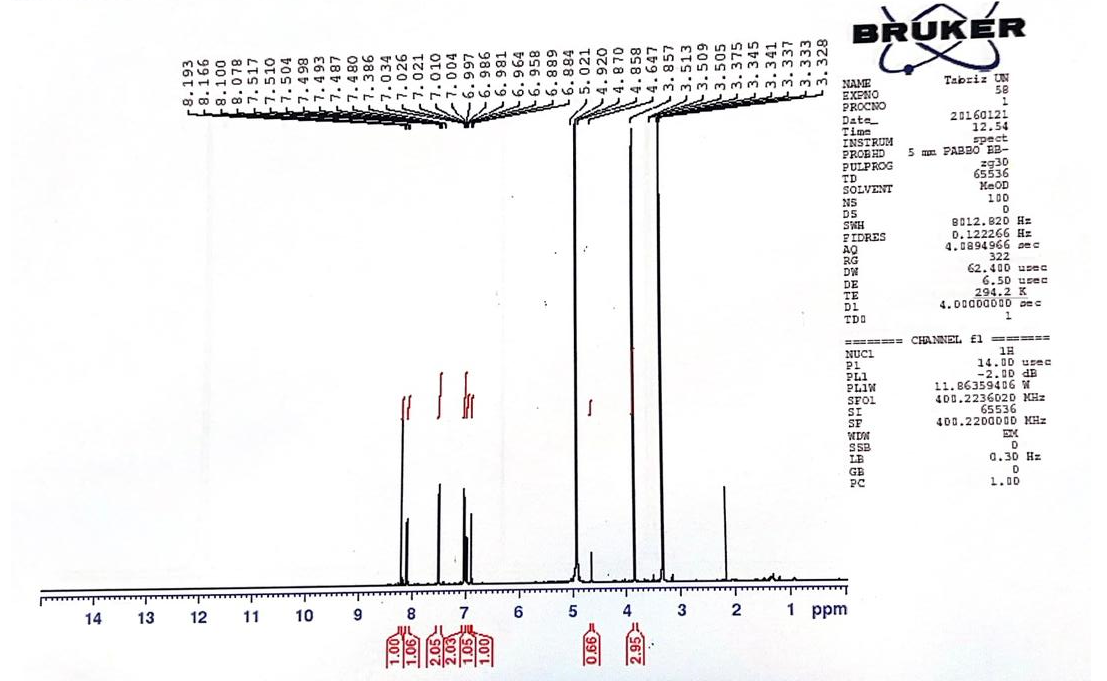


**Supplementary file 1C**. Nuclear magnetic resonance (NMR) spectra of formononetin purified from *Astragalus onobrychis* L.


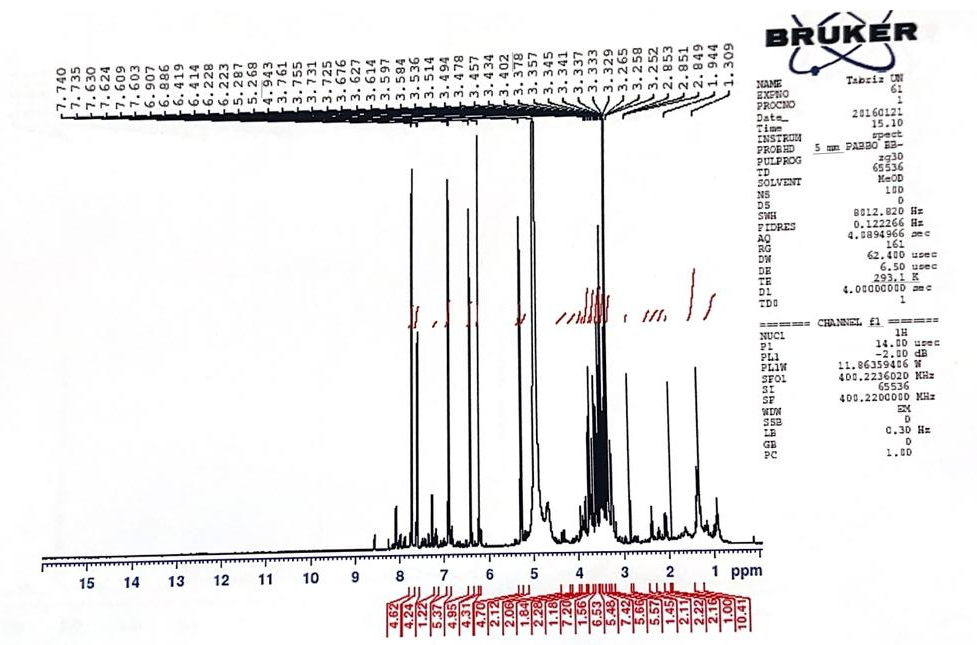


**Supplementary file 1D**. Nuclear magnetic resonance (NMR) spectra of isoquercitrin purified from *Astragalus onobrychis* L.


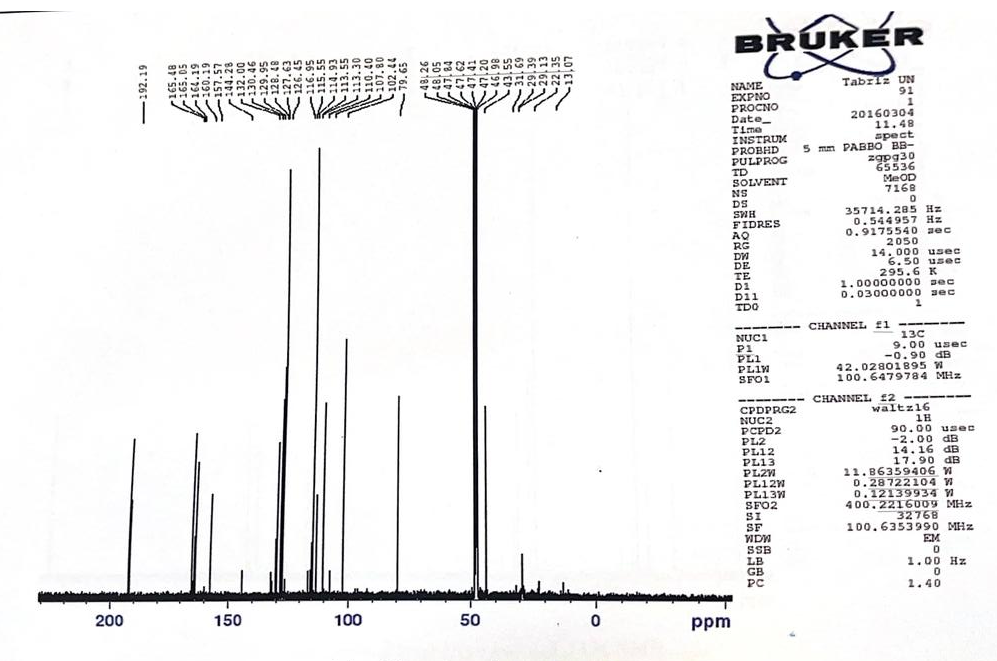


**Supplementary file 1E**. Carbon-13 nuclear magnetic resonance (13C-NMR) spectra of liquiritigenin purified from *Astragalus onobrychis* L.


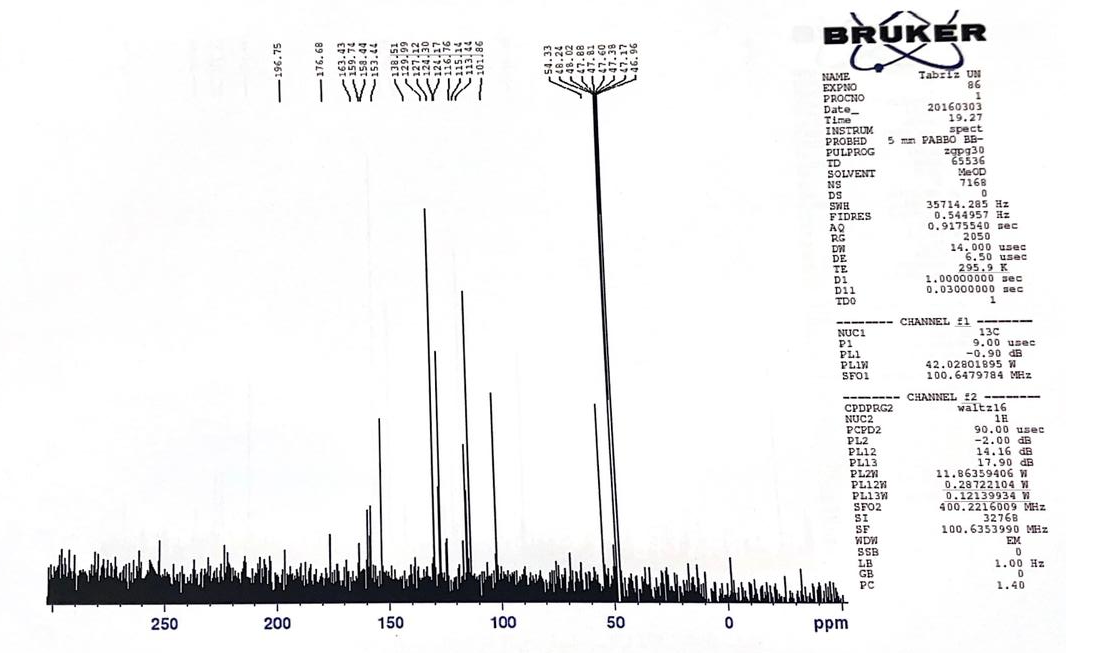


**Supplementary file 1F**. Carbon-13 nuclear magnetic resonance (13C-NMR) spectra of formononetin purified from *Astragalus onobrychis* L.


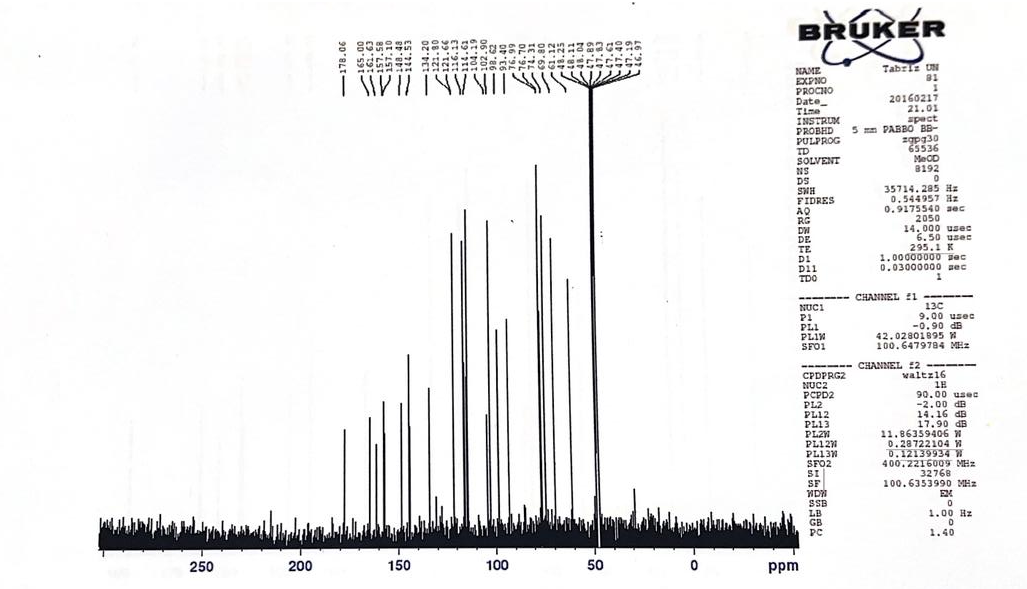


**Supplementary file 1G**. Carbon-13 nuclear magnetic resonance (13C-NMR) spectra of isoquercitrin purified from *Astragalus onobrychis* L.
